# Effect of an antioxidant on the microtensile bond strength (µTBS) of restored teeth after dental bleaching

**DOI:** 10.1080/07853890.2021.1897310

**Published:** 2021-09-28

**Authors:** Inês Caetano Santos, Rodrigo Abreu, Luís Proença, António H. S. Delgado, Mário Polido, José João Mendes

**Affiliations:** a Centro de Investigação Interdisciplinar Egas Moniz (CiiEM), Egas Moniz Cooperativa de Ensino Superior, Caparica, Portugal; b Instituto Universitário Egas Moniz (IUEM), Egas Moniz Cooperativa de Ensino Superior, Caparica, Almada, Portugal

## Abstract

**Introduction:**

Dental bleaching is regarded as a safe medical treatment for those who want to achieve a brighter smile. Bleaching is a chemical process in which oxidation occurs, affecting the way teeth absorb or reflect light [1]. Oxidative ability of bleaching agents and the presence of free hydroxyl radicals in the apatite modifies the mineral and protein composition of the enamel increasing its solubility. This renders the enamel surface adverse to the best bonding conditions [2,3]. Some strategies, including antioxidants such as sodium ascorbate, are capable of re-establishing the bond strength to nearest normal values, showing to be an effective method when used immediately after dental bleaching treatment [4,5]. The purpose of this study is to evaluate the effect of an antioxidant on the microtensile bond strength of bleached teeth that were subsequently restored, comparing different waiting times for the procedure.

**Materials and methods:**

Thirty human permanent molars were sectioned into halves (*n* = 60), and randomly distributed between three groups: control (CG), bleaching (G1) and bleaching + sodium ascorbate (G2). Groups G1 and G2 were bleached 2 h/day for a 7-day period. After bleaching, G2 received a 10% sodium ascorbate gel for 50 min. In each group, samples were divided in equal parts where one part (*n* = 5) was immediately restored with an adhesive system and a resin composite (T0) and the other half (*n* = 5) was stored, in artificial saliva at 37 °C, and restored after 7 days (T1). After 24 h, samples were sectioned into microspecimens (∼1mm^2^) and tested at a crosshead head-speed of 0.5 mm/min. Data analysis was performed using a factorial ANOVA, at a significance level of 5%.

**Results:**

Groups in which sodium ascorbate was applied presented mean µTBS values (G2T0: 22.1 ± 3.3 MPa; G2T1: 24.5 ± 2.9 MPa) significantly higher than the bleaching only and immediately restored group (G1T0, 10.9 ± 3.3 MPa) (*p* < .001). The group in which teeth were beached and restored after 7 days (G1T1, 19.4 ± 2.6 MPa) showed significantly higher values than the group immediately restored after bleaching (G1T0) (*p* = .006). No significant differences were found between the groups in which sodium ascorbate was applied (G2) and the group in which teeth were bleached and restored after 7 days (G1T1) (*p* > .05).

**Discussion and conclusions:**

Sodium ascorbate is capable of capturing free oxygen radicals retained on the enamel. These promote decomposition of hydrogen peroxide, which in turn prevents the complete polymerisation of the adhesive system thus affecting bond strength [2,5]. The application of sodium ascorbate is an effective alternative to improve bond strength when compared to a waiting period before the restorative procedure.
